# DRGKB: a knowledgebase of worldwide diagnosis-related groups’ practices for comparison, evaluation and knowledge-guided application

**DOI:** 10.1093/database/baae046

**Published:** 2024-06-06

**Authors:** Shumin Ren, Lin Yang, Jiale Du, Mengqiao He, Bairong Shen

**Affiliations:** Department of Pharmacy and Institutes for Systems Genetics, West China Hospital, Sichuan University, Frontiers Science Center for Disease-Related Molecular Network, Xinchuan Road 2222, Chengdu 610041, China; Department of Computer Science and Information Technology, University of A Coruña, Faculty of Infomation, Campus of Elvina, A Coruña 15071, Spain; Department of Pharmacy and Institutes for Systems Genetics, West China Hospital, Sichuan University, Frontiers Science Center for Disease-Related Molecular Network, Xinchuan Road 2222, Chengdu 610041, China; Department of Pharmacy and Institutes for Systems Genetics, West China Hospital, Sichuan University, Frontiers Science Center for Disease-Related Molecular Network, Xinchuan Road 2222, Chengdu 610041, China; Department of Pharmacy and Institutes for Systems Genetics, West China Hospital, Sichuan University, Frontiers Science Center for Disease-Related Molecular Network, Xinchuan Road 2222, Chengdu 610041, China; Department of Pharmacy and Institutes for Systems Genetics, West China Hospital, Sichuan University, Frontiers Science Center for Disease-Related Molecular Network, Xinchuan Road 2222, Chengdu 610041, China

## Abstract

As a prospective payment method, diagnosis-related groups (DRGs)’s implementation has varying effects on different regions and adopt different case classification systems. Our goal is to build a structured public online knowledgebase describing the worldwide practice of DRGs, which includes systematic indicators for DRGs’ performance assessment. Therefore, we manually collected the qualified literature from PUBMED and constructed DRGKB website. We divided the evaluation indicators into four categories, including (i) medical service quality; (ii) medical service efficiency; (iii) profitability and sustainability; (iv) case grouping ability. Then we carried out descriptive analysis and comprehensive scoring on outcome measurements performance, improvement strategy and specialty performance. At last, the DRGKB finally contains 297 entries. It was found that DRGs generally have a considerable impact on hospital operations, including average length of stay, medical quality and use of medical resources. At the same time, the current DRGs also have many deficiencies, including insufficient reimbursement rates and the ability to classify complex cases. We analyzed these underperforming parts by domain. In conclusion, this research innovatively constructed a knowledgebase to quantify the practice effects of DRGs, analyzed and visualized the development trends and area performance from a comprehensive perspective. This study provides a data-driven research paradigm for following DRGs-related work along with a proposed DRGs evolution model. Availability and implementation: DRGKB is freely available at http://www.sysbio.org.cn/drgkb/.

**Database URL**: http://www.sysbio.org.cn/drgkb/

## Introduction

Diagnosis-related groups (DRGs) were originally conceptualized and developed in the USA, with the fundamental aim of categorizing cases based on the similarity of their clinical treatment pathway and resource usage (1), and then used for prospective payment system. With the continuous growth of healthcare expenditures in most countries in recent years (2), and inpatient costs being the largest single component, the burden of cost control has increasingly shifted towards expenses incurred by inpatients (3). The DRGs payment system is believed to reduce inpatient costs and enhance the efficiency of medical resource utilization compared to fee-for-service payments. Meanwhile, DRGs are valuable tools in medical policy making as it’s supposed to promote cost-effective and high-quality care, support data-driven decision-making and contribute to the ongoing evolution of healthcare payment and delivery systems. Their importance lies in their ability to align financial incentives with desired healthcare outcomes. Therefore, since its inception, DRGs has been widely adopted globally and has been subject to numerous applications and practices. However, the effects of DRG-based prospective payment system implementation in different countries are not the same. Common outcome measures to evaluate the effectiveness of DRGs practice include mortality, readmissions, average length of stay (ALOS), cost per case, reimbursement rate, explanation of cost variances and so on (4–6). But no research has yet collected data on these practices on a large scale, quantified the effects and explored the underlying reasons.

The Centers for Medicare and Medicaid Services (CMS) launched the value-based purchasing program (VBP), which aims to encourage healthcare providers to provide high-value health services through a series of supporting policies and principles (7). This series of actions emphasizes that on the premise of controlling medical costs and ensuring medical efficiency, DRGs pay more and more attention to ‘value of medical practice’ during its development, which also means that indicators such as medical quality and medical services will also perform as an important role in the evaluation of DRGs practice (8). Therefore, integrating a reasonable DRGs practice evaluation index system is an urgent requirement in the current era of value-based medicine. At the same time, there are currently a large number of literatures about the practice of DRGs across the country, which have potential value for DRGs policy formulation. Ginsburg and Phillips (9) also brought forward that refined health data systems and medical reimbursement policies are essential for the implementation of precision medicine. Knowledgebase can store information in a structured and explainable way (6). As a result, as a tool for integrating multidimensional information and providing decision-making suggestions, it affords an effective and important basis for medical policy making in the precision medicine era. Therefore, in this study, we established the first open accessed evidence-based DRG practice knowledgebase (DRGKB), incorporating a large amount of empirical evidence on the impact of DRG introduction. Through the knowledge base, we visualized and classified the practice data, proposed a comprehensive scoring method for assessing the impact of DRGs practice, and made discussion and analysis about the emerging status quo. Meanwhile, it is believed that models based on precision medicine we proposed in the end can better map real disease status of patients into reasonable individual medical expenses in the future. In conclusion, the establishment of DRGKB and the conception of new evolutionary model provide groundbreaking indications for the development of future DRGs.

## Methods

### Data collection

All data for DRGKB were manually collected from the public database PubMed (www.ncbi.nlm.nih.gov/pubmed). In order to ensure that no literature that may fit the theme of this study is missed, our study adopts the principle of ‘first large-scale search, then gradually screening out’ to include literature. The specific rules are as follows.

We conducted literature search using ‘Diagnosis-Related-Group*[Ti] OR DRGs[Ti]’ as terms. We set the following inclusion and exclusion criteria (i): the topics of the research should be related to the development, implementation, refinement, assessment and validation of DRGs (2). The research should be carried out on a representative population sample and provide comprehensive assessment indicators and associated outcomes, such as average length of stay (ALOS), mortality rates, readmission rates, etc.; or the research suggests novel approaches to refine the grouping principles or cost prediction ability of DRGs, such as incorporating new clinical or demographic factors into the grouping algorithm, or developing new risk adjustment models, etc. To give a more intuitive example, literature with titles similar to the long-run effects of DRG payment on hospital lengths of stay in a publicly funded healthcare system (10), or improving the clinical ability and quality of endocrinology department with DRGs tool (11), will be included in our scope of inclusion (3). Use proper statistical or research methods, and the conclusions drawn in the article must be supported by evidence (4). Certain types of articles were excluded, such as reviews, commentaries, letters, articles without full-text access and those not written in English.

**Figure 1.**
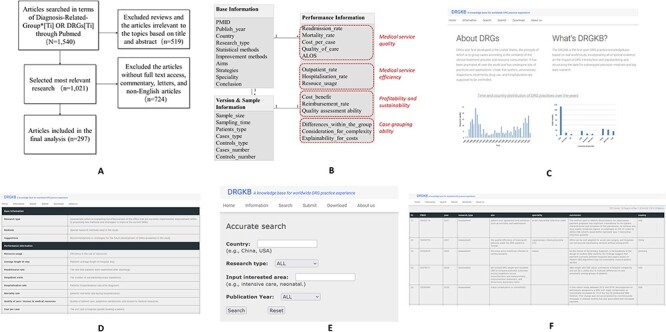
Work flow of the study and the website interfaces of DRGKB (A) Flowchart of the search strategy (B) Entity-relation diagram of DRGKB, where the red parts represent the classifications of indicators; the interface of DRGKB (C) Home, (D) Information page, (E) Search page and (F) Search results page.

Based on these criteria, we acquired 1540 articles on PubMed with search terms by 1 June 2022. Irrelevant studies, such as dorsal root ganglia (DRGs) which share the same abbreviation but are unrelated concepts, were first excluded. After that, since our database only collected evidence-based practice related to DRGs assessment and improvement, we excluded qualitative DRGs studies based on subjective judgments (*n* = 125), epidemiological studies using DRGs data (*n* = 264) and reviews (*n* = 42). We then removed letters and comments (*n* = 39), articles without full text (*n* = 254) and non-English articles (*n* = 431). At last, a total of 297 studies were obtained. The selection process for literature collection is outlined in Figure 1A.

### Website construction and knowledgebase constitutions

We created the website (http://sysbio.org.cn/DRGKB/) via WAMP (Windows + Apache + MySQL + PHP), and the client side is written in HTML and CSS scripting language. The four functions of information introduction, data retrieval, information submission and data sharing are achieved. The database contains three tables, which are Base Information Table, Performance Information Table and Version and Sample Information Table. The entity-relation diagram is illustrated in Figure 1B. In the Performance Information Table, which represents the architecture for evaluating DRG classification system performance and the impact of DRG-based prospective payment system, the table categorizes evaluation indicators and defines the criteria for assessing indicator performance. For each evaluation indicator in the Performance Information Table, its content consists of ‘+’ or ‘−’ and the corresponding original text. The symbol ‘+’ indicates that the practice has positive effects on the index or the index has risen; ‘−’ indicates that the practice has negative effects on the index or the index has declined. The text from the original text specifically describes the performance of the indicator.

### Data analysis and comprehensive scoring

Based on the collected data of DRGKB, we carried out descriptive statistics and analysis on statistics methods, outcome measurements performance, improvement strategy and specialty performance. American scholar Christopher Moriates suggested DRGs of value-based medical system should be evaluated from three dimensions, that is, quality, cost and patient experience (12). Additionally, the core assessment indicators proposed by China CHS-DRG include medical service capacity, medical service quality, medical service efficiency, medical service safety and medical cost control (13). Referring to the above principles and the indexes appearing in the collected literature of knowledgebase, we divided the evaluation indicators into four categories: (i) medical service quality; (ii) medical service efficiency; (iii) profitability and sustainability; and (iv) case-grouping ability. Among them, (i)–(iii) focus more on the medical and financial impacts of the DRG-based prospective payment system on hospitals, while (4) focuses on the rationality of the DRG classification system and its applicability in practical implementation. Quantitative statistics and scoring were made on the results of each type of evaluation indicators. For these four categories of indicators, the scores are calculated using the following formula.


$${S_j} = \frac{\sum \left( {n{{(p)}_i}/{n_i}} \right)}{i}$$

In the formula, where *i* represents the number of sub-indicators in each category, *n_i_* represents the total research number for each sub-indicator and *n*(*p*)*_i_* represents the research number with positive evaluation results for each sub-indicator. For example, in the category of medical service quality, a decrease in readmission rate indicates a positive evaluation result. *S_j_* represents the evaluation scores for the four different indicator categories. After obtaining the evaluation scores for each category, summing up the values of *S_j_* and subsequently dividing by the total number of indicator categories, 4, yields the final comprehensive evaluation score. This can be expressed as follows.


$${S_{total}} = \frac{\sum \left( {{S_j}} \right)}{4}$$

The scores range between 0 and 1, with higher scores indicating a more positive impact of DRGs, while lower scores suggest a more negative influence.

For each category of indicators, corresponding motivations were analyzed through text mining. After that, research of improvement type was analyzed to investigate the current development trend of DRGs. Meanwhile, the most concerned areas were analyzed based on their distribution and evaluation performance.

## Results

### Knowledgebase modules and functions

DRGKB is available at http://sysbio.org.cn/DRGKB/. Figure 1(C–F) displays the interfaces of DRGKB. The homepage of DRGKB is mainly about the introduction of DRGs, the aim of the website and statistics of related entries. In the information page, the entity-relationship and the explanations of attributes contained in tables are displayed, which provides help for following retrieval. The search page sets several key search criteria, including country, research type, specialty of interest and published year. Combination searches can be performed to meet the needs of users, while options or hints are provided to conduct efficient searches. The search results page displays the main information for each entry based on the search terms

### Overview and analysis of the results of statistics methods and outcome measurements

**Figure 2.**
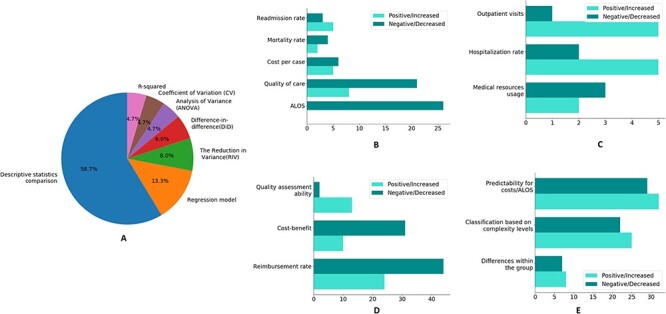
(A) The descriptive statistics of statistical methods of research. The performance of four different types of indicators. (B) Medical service quality; (C) medical service efficiency; (D) profitability and sustainability; (E) case grouping ability.

Firstly, we conducted statistical analysis and presented statistical methods that were used more than five times, as shown in Figure 2A. Descriptive statistics comparison is the most commonly used statistical method, where studies often use descriptive statistics to analyze and compare the changes in outcome indicators between the control group and the intervention group or among time series. For continuous variables, the data were summarized using the mean and standard deviation, while categorical variables were presented as frequencies or percentages, and significance tests were performed to compare results between groups. This method facilitates researchers in comprehending the relationships, trends and differences among variables, thereby effectively elucidating the characteristics of the data. Regression analysis is employed to estimate the impact of interventions on outcome variables by adjusting for single or confounding factors, such as identifying the factors that determine the profitability of DRGs (14). Through regression analysis, researchers can explore the associated influencing factors, estimate model parameters and standard errors. The *R*^2^-value is utilized to explain the fitness of the regression model. Difference-in-differences (DiD) analysis is often used to evaluate the impact of policies or intervention measures on observed data. For instance, DiD can be employed to examine the effects of introducing DRGs on variables like length of hospital stay, number of diagnoses and primary diagnosis outcomes (10, 15, 16). Compared to descriptive statistics, regression analysis and DiD can provide causal relationships and inferential conclusions. ANOVA, the reduction in variance (RIV) and coefficient of variation (CV) are often employed to assess differences within or between groups in DRGs classification system, such as length of hospital stay and cost per case, contributing to gauging the level of homogeneity or heterogeneity within and between DRGs, thereby determining the effectiveness of DRGs classification (17–19). From above, we can conclude that the abundant statistical and research methods used for different research objectives exhibits the knowledgebase’s reliability. For the indicators for four categories, we have listed the definitions and criteria for assessing indicator performances, as described in Table 1, and based on which, we have conducted the analysis of the descriptive statistical results.

**Table 1.** The classifications, definitions and criteria for assessing indicator performances

Indicator classifications	Indicator performance	Definitions
Medical service quality	Length of stay ±	Compared with the control groups or among different time-series groups, the length of stay of the study group shows a statistically significant increase or decrease in number or trend.
	Readmission rate ±	Compared with the control groups or among different time-series groups, the readmission rate of the study group shows a statistically significant increase or decrease in number or trend.
	Cost per case ±	Compared with the control groups or among different time-series groups, the cost per case of the study group, including but not limited to drug cost, material cost, examination cost, etc. shows a statistically significant increase or decrease in number or trend.
	Quality of care ±	Compared with the control groups or among different time-series groups, there are statistically significant changes in the study group regarding to medication specifications, hospitalization procedures, surgical indications, innovative procedures acceptance, patient satisfaction, access to medical resources, etc. qualitatively or quantitatively.
	Mortality rate ±	Compared with the control groups or among different time-series groups, the mortality rate of the study group shows a statistically significant increase or decrease in number or trend.
Medical service efficiency	Outpatient visits ±	Compared with the control groups or among different time-series groups, the outpatient visits of the study group show a statistically significant increase or decrease in number or trend.
	Hospitalization ±	Compared with the control groups or among different time-series groups, the hospitalization rate or hospital admissions of the study group show a statistically significant increase or decrease in number or trend.
	Resource usage ±	Compared with the control groups or among different time-series groups, the bed occupancy, staff number, bed days, cases number, time efficiency index (TEI), case-mix index (CMI), risk weight (RW), surgery utilization of the study group shows a statistically significant increase or decrease in number or trend.
Profitability and sustainability	Cost benefit ±	Compared with the control groups or among different time-series groups, the financial balance, financial balance as percentage of expenditure, marginal cost, cost efficiency index (CEI) of the study group shows a statistically significant increase or decrease in number or trend.
	Reimbursement ±	Compared with the control groups or among different time-series groups, the reimbursement charge, the proportion of cost and reimbursement charge, the amount of out-of-pocket (OOP) payments and OOP payments as a share of total inpatient expenditure of the study group shows a statistically significant increase or decrease in number or trend.
Case grouping ability	Differences within the group ±	Within the same DRG group, the length of stay, cost or other patient characteristics, are statistically significantly the same or different.
	Differences between the groups ±	Among different DRG groups, the length of stay, cost or other patient characteristics, are statistically significantly the same or different.
	Consideration for classification complexity ±	In DRG classification system, whether the complications and comorbidities of the disease, severity, risk of death, surgical procedures and other complex adjustment factors are considered.
	Explainability for costs ±	Whether the complex adjustment factors of DRG groups have statistically significant correlation or causal relationship with responding cost, length of stay, resource utilization and reimbursement.

#### Medical service quality

In Figure 2B, 26 evidence showed that ALOS decreased after the introduction of DRGs, largely because the compensation mechanism of DRGs would promote the improvement of service efficiency (20), including the implementation of clinical pathways and the improvement of medical technology (15, 21), thus incentivizing hospitals to generate surplus and reduce unit cost (22). For cases where cost has increased, this is partly due to insufficient reimbursement after the implementation of DRGs (23, 24).

**Figure 3.**
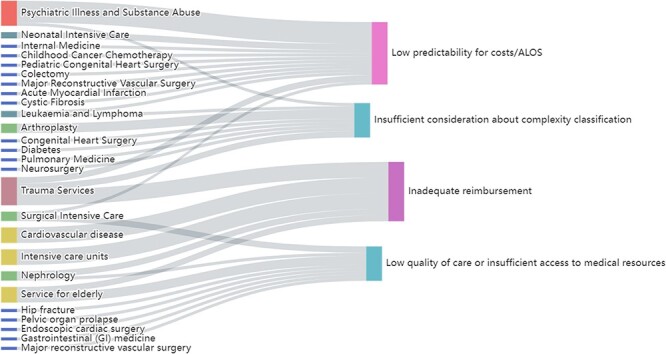
Sankey figure of specialties distribution of low predictability for costs/ALOS, low quality of care or insufficient access to medical resources, insufficient consideration about complexity classification and inadequate reimbursement, respectively.

However, assessing the impact of DRG payment system solely based on ALOS and cost per case is insufficient as it overlooks the effects on medical quality. DRGs can reduce overtreatment, promote clinical pathway implementation and enhance medical efficiency (25). It also requires doctors to improve clinical service capabilities, surgical quality and postoperative care quality (20). Additionally, DRGs can incentivize hospitals to improve their medical quality and efficiency (26). Nevertheless, certain specialties and treatments may not receive sufficient reimbursement, which limits patients’ access to these medical services and hinders technological innovation and medical quality improvement. Moreover, 21 pieces of evidence indicate that DRGs may lead to a decline in the quality of care. This could be attributed to the fact that reducing LOS and operation rates can negatively affect the quantity and quality of care (27, 28), leading to a downward trend in medical opportunities, particularly for geriatrics, surgical ICU and other high-risk patients. Figure 3 demonstrates the specialties distribution of specific performances using Sankey figure, which visualizes the classification and statistics of above findings. In addition, regarding indicators such as readmission rate, mortality rate and cost per case, there is a roughly balanced distribution of both positive and negative evidence. This indicates that the impact of DRGs in various countries and domains cannot be universally generalized in these aspects.

#### Medical service efficiency

DRGs have different effects on resource use efficiency as shown in Figure 2C. On the one hand, the incentive system of DRGs can reduce over-treatment by changing the behavior of doctors (, 29), improve the bed turnover rate and utilization rate (20), and improve service efficiency (25, 30, 31). However, fixed DRGs rates, while having a positive effect on cost control, limit the incentives for resource utilization (32, 33). In terms of hospitalization rate, several evidence show that the introduction of DRGs has brought about an increase in hospitalization rate. Retrospective payment systems based on DRGs link hospital payments to the number of patients they serve, incentivizing providing more medical services (34). In addition, with the popularization of clinical pathways, the quality of surgery has improved, increasing the hospitalization rate (11). However, the increased hospitalization rate may also be a result of up-coding under reimbursement incentives (35, 36). On the other hand, since DRGs payment system reimburses inpatient medical services for each DRG at a fixed rate, hospital can transfer inpatients to outpatient settings to shift medical costs (37). This phenomenon may lead to an increase in outpatient visits and unnecessary resource usage (38–41).

#### Profitability and sustainability

In Figure 2D, there are 44 evidence showing insufficient reimbursement rates exist. We conducted statistics on the areas involved in the Figure 3, and found that the cardiovascular diseases, trauma and ICUs have the most cases of insufficient reimbursement rates. After that came elderly services and nephrology department. This is mainly due to differences in the complexity of case conditions and care, and the fact that the current DRGs still does not reflect human resources and service costs well (42, 43). It is worth noting that 31 evidence shows that DRGs brought about a reduction in hospital cost-benefit. The arbitrary DRG pricing method without considering local situation (44, 45), and failure to take into account the complexity and heterogeneity of patients in the same group (46), can lead to economic inhibition. In addition, from the perspective of quality assessment, several evidences suggest that DRGs can be a useful tool to assess differences in the complexity of patients’ conditions and care among groups.

#### Case grouping ability

Figure 2E displays the differences within the DRG group, regarding of which, large inter-individual variances could exist in specific domains. Using tailored patterns for disease grouping can greatly reduce within-group differences. For example, Montefiori *et al*. adjusted for birth weight through cluster analysis (47), while Endrich integrated gestational age into grouping variables (48). Meanwhile, certain evidence indicates that there is minimal variation among DRG groups (49, 50), which highlights the need for decision-makers to use higher-quality data and consider more refined grouping logic when formulating DRG classifications. Figure 2E also displays some areas lack comprehensive consideration of complexity, complications and comorbidities (C&Cs), leading to inaccurate case classification. Luo *et al*. established six hospitalization expenses of senile cataract patient (HECP) standards using the E-CHAID algorithm (51), while the establishment of Aristotle score assigns DRG and cost-weight values to better evaluate surgical performance complexity (52–55). Machine-learning-based cost calculation methods, such as random forest (56), have the advantages of high transparency and efficiency for cost interpretation and LOS prediction (57, 58). Nevertheless, 29 evidence reports that DRGs do not explain costs well. Figure 3 displayed the areas where cost interpretation performed poorly. Most evidence was found to be related to psychiatric problems and substance abuse, followed by trauma services and neonatal ICUs. The statistics of specialties distribution of specific indicators are given in Supplementary Appendix Table.S1.

#### Scoring results

According to the ‘Methods’ section, we calculated scores for each category of indicators and their overall scores, as detailed in Table 2. The final comprehensive score is 0.535, indicating that the impact of DRGs on the hospital operation in different countries is mixed. Therefore, specific analyses are required to comprehensively evaluate the positive and negative effects of DRGs globally.

**Table 2.** The comprehensive assessment results of the impact of DRGs

Categories	Score
Medical service quality	0.572
Medical service efficiency	0.573
Profitability and sustainability	0.487
Case-grouping ability	0.507
Total score	0.535

#### Case study

In addition to the above data statistics and analysis on outcome measurements, we have also selected several cases collected in DRGKB to provide a more specific explanation and presentation of the positive impacts and shortcomings of DRGs on hospital operations.

For example, in the study by Ma and Wang (20), 1200 admitted patients were recruited and grouped into internal medicine, surgery and intervention groups. Case mix index and costs were then calculated. It was found that DRG management, by reducing the length of hospital stays, guiding healthcare professionals to focus on surgical quality, and accelerating postoperative recovery, improved the clinical service capacity of the hospital. Simultaneously, DRG management required physicians to pay more attention to surgical quality, increasing surgical turnover and bed utilization rates. Real-time reminders to physicians, based on disease types and economic conditions, helped control costs, thereby constraining total patient medical expenses and saving healthcare resources. DRG management also demanded physicians to conduct relevant assessments based on patient conditions, increasing the safety of medical activities and strengthening healthcare quality. By accelerating postoperative recovery, reducing hospital costs and improving the quality of medical services, DRGs ultimately enhanced patient satisfaction. Busse *et al*. (22) reviewed the experiences with DRG systems in 12 European countries. They found that in the USA, the DRG payment system helps control healthcare costs by setting fixed payment amounts, thereby limiting the expenses for services. In Europe, particularly in England, the DRG payment system has encouraged more day care activities, resulting in an overall increase in healthcare activities. This may be beneficial for enhancing service flexibility and meeting patient needs. However, in some European countries, the uncertainty remains regarding whether the increased activity leads to higher hospital costs. Meanwhile, a study in Sweden found that excessively shortening hospital stays could lead to a decline in service quality. Studies in some other European countries suggest that DRG payment systems have not significantly altered mortality and readmission rates. DRG payments may also lead to unintended consequences, such as cherry-picking, upcoding, overtreatment and high readmission rates (59). Lee *et al*. (39) collected extensive claims data in South Korea to study the impact of DRG policy implementation on different types of healthcare institutions and medical conditions. Consistent with studies in the USA, Switzerland, Taiwan and elsewhere, they found a significant reduction in hospitalization days across healthcare institutions after the implementation of DRGs. However, the implementation of DRGs resulted in a significant increase in outpatient visits within the 30 days prior to hospitalization. This may be attributed to healthcare institutions shifting medical costs to outpatient services not covered by DRGs, creating the so-called ‘balloon effect’. Yuan *et al*. (32) took acute myocardial infarction (AMI) as a case study, selecting 2895 AMI patients from two major tertiary hospitals in China to assess the policy’s impact. They found that the DRG payment system effectively controlled medical expenses for AMI patients. Following the implementation of DRGs, the use of percutaneous coronary intervention (PCI) significantly increased, primarily due to the rapid advancement of interventional cardiology techniques in China. However, despite a substantial increase in PCI use, there was a significant decrease in PCI utilization for the intervention group, potentially linked to a weakened incentive for physicians to provide PCI services for profit. Additionally, medical expenses significantly increased for uninsured patients, suggesting that the implementation of DRGs may lead to a shift in medical costs from insured to uninsured patients.

In conclusion, DRGs, as a healthcare payment system, have achieved some successes in improving efficiency and cost control. However, they also face challenges and potential negative impacts in different countries and situations, necessitating continuous refinement and adjustments in specific practices.

### Promotion strategy and analysis of different specialties

#### Analysis of research for improvement

A total of 58 studies have proposed specific methods to improve DRGs. Through our observation and statistics, we found that since 2000, as many as 13 studies have used big data or machine-learning methods to systematically improve or refine DRGs. Table 3 summarizes these DRG studies involving model construction or advanced algorithms. These studies reflect the recent focus on data-driven methods for DRGs.

**Table 3.** Summary of DRGs-related big data or machine-learning research

PMID	Specialty	Year	Methods
34 917 572	NA	2021	Reasonable pricing is given through the game pricing model to prevent adverse selection problems in the operation of the DRGs system.
34 753 472	NA	2021	Proposed a data-based and machine learning-based grouping method, which can be trained by real cases or even simulated cases.
32 785 282	Neonatal intensive care unit	2020	With cluster analysis and Tobit regression, the main cost determinants of neonatal respiratory distress syndrome were taken into account: birth weight, gestational age and discharge status, thereby estimating the total cost per patient per day.
31 484 551	NA	2019	Applied data detection algorithms to predict medical expenses.
31 230 955	Conversion total knee arthroplasty	2019	Multiple regression analysis was performed to identify independent risk factors for primary TKA and those who underwent conversion TKA.
29 503 750	Spinal fusion	2018	Compared the performance of machine learning methods for predicting the profit or loss performance of spinal fusion.
29 487 824	Senile cataract	2018	Used the exhaustive Chi-squared automatic interaction detector (E-CHAID) model to analyze the influencing factors of senile cataract.
26 880 102	NA	2016	Used a graph-based clustering algorithm to identify the DRG groups with similar production profiles.
25 834 619	Chronic obstructive pulmonary disease	2015	Using conditional linear regression, the best model for predicting readmission was determined with the highest c-statistic.
24 519 749	NA	2015	Used a fixed-effects three-stage least squares to investigate to which extent DRG prompts hospitals to adjust output mix to make profits.
15 531 958	NA	2004	Function-linked variables were added to multiple linear regression models to measure the ability of DRGs to explain LOS variances.
15 481 636	NA	2004	Through a systematic search algorithm, it established a benefit calculation program with more than 2000 comorbidities to calculate the additional compensation a specific DRG could receive.
15 466 092	Children care	2004	Used large comparison databases to determine the measurements of severity.

#### Performance analysis of specialties

We counted the areas of concern for each research and listed the specialties statistic with more than two occurrences in Supplementary Appendix Table S2. The most concerned area was found to be cardiovascular diseases with 20 studies, ICU (including neonatal ICU) with 14 studies and fractures & arthroplasty with 10 studies, as well as mental health and acute care.

The cardiovascular field includes various diseases, such as AMI, congenital heart surgery (CHS), surgical aortic valve replacement (SAVR) and heart failure. Due to the high morbidity and costs of AMI and underfunding of other cardiovascular procedures (42, 60, 61), research in this field is intensive. Some evidence shows that the introduction of DRG reduced patient length of stay and average cost of services, but increased readmission rates and mortality for AMI patients due to reduced medication or cardiac catheterization (60, 62, 63). For patients with chronic heart failure, there is a risk of premature discharge and increased readmission rates (62). In addition, DRG reimbursement did not reflect true costs and resulted in economic disadvantage and limitations in service delivery (61, 64, 65). However, the creation of the Aristotle score in G-DRG is a good demonstration for DRG improvement in the cardiovascular field, which assigns reasonable surgical complexity and cost weights as the basis for reimbursement, ensuring the rationality of grouping (52–54). Other studies found that disease severity measured by specific clinical criteria [e.g. myocardial enzyme peak levels (66)] and post-admission diagnoses were effective prediction factors for AMI mortality and cost (67, 68). Overall, more research is needed to improve DRG reimbursement and prediction factors in the cardiovascular field.

ICUs have become a challenging area since the introduction of DRGs due to their high costs, limited beds and intensive medical services (69). High out-of-pocket payments create social and financial problems for patients. The complexity and mortality in ICUs vary greatly (70), especially for neonates, which should consider factors such as gestational age, birth weight and complications (47). Evidence from 1985 to 2004 shows that ICU DRGs were under-reimbursed (71–75), limiting the provision of medical resources for neonates and elderly patients. DRG also cannot reasonably explain generated costs or LOS, which are affected by assisted ventilation, surgery, survival and mode of discharge (76–78). Thus, the key to the long-term stable development and profitability for ICUs is to formulate reasonable severity adjustors, such as the All Patient Refined-Diagnosis Related Group (APR-DRG) Risk of Mortality Score (79). Research for improvement suggests that comprehensive indicators, including birth weight for neonates, need to be considered urgently in the future (47, 78).

In the fracture and arthroplasty fields, the use of arthroplasty for the treatment of degenerative lesions and trauma indications has increased rapidly over time, making this procedure an important focus area for prospective payment model (46). Meanwhile, the etiologies, indications and complications of total knee arthroplasty and total hip arthroplasty are diverse (80, 81). Furthermore, hip fractures are common among older adults and are a major public health concern worldwide (82). One evidence showed that DRG implementation significantly increased the prescription rate of analgesic and psychotropic medications for hip fracture care, possibly due to short post-operative recovery period (83, 84). Three evidence suggests the lack of risk adjustment for C&Cs for joint replacement and joint infection resulted in a strong economic disincentive (46, 85, 86). Therefore, some studies proposed that for specific situations, such as C&Cs of conversion total knee arthroplasty and knee replacement, independent DRG codes and more comprehensive groupings are required to cover costs (80, 87).

## Discussion

While the basic concept of DRGs remains consistent, there can be variations in their practices across different countries or regions due to differences in healthcare systems, payment models and regulatory frameworks. In 1976, DRGs was first developed in USA. In 1981, refine DRGs were successfully developed, which had incorporated complications and comorbidities (C&Cs), and included new information such as patients’ outcomes and admission methods (88). From 1987 to 1991, All Patient DRGs (AP-DRG) and All Patient Refined DRGs (APR-DRG) were subsequently developed, which modified the classification of newborns and HIV infection, added more comprehensive considerations for grouping based on C&Cs and age with disease severity and mortality risk (89). APR-DRG takes into account the second diagnosis (additional diagnosis) and its interaction with others during the grouping process, which was more suitable for older age insurance, obstetrics, pediatrics and neonatology (26, 90). The intensive attention and research on DRGs in USA at this stage provided an important reference for the subsequent development of DRGs in other countries. From 2000 to 2010, DRGs have been popularized in most western countries, mainly in the USA, Australia and Germany. In this period, Australia introduced APR-DRGs (91), while the local version of DRGs, AN-DRGs (Australia National DRGs) was also developed (1992), which was subsequently replaced by more advanced AR-DRGs (Australian Refined Diagnosis Related Groups). After continuous revision and improvement, Australia updated the AR-DRG with the Patient Clinical Complexity Level (PCCL). Combined with the Clinical Complexity Level (CCL) per case, the impact of multiple C&Cs on resource use is quantified (92). Meanwhile, the Episode Clinical Complexity Model was introduced with the emphasis on additional diagnoses (93). Additionally, DRGs are also widely used in the private sector in Australia (94). European countries often have their own DRG systems tailored to their specific healthcare contexts. For example, Germany uses the German DRG system (G-DRG) (95). Germany developed G-DRGs (Germany DRGs) based on AR-DRGs. Germany adopted supplementary fees to compensate reimbursement for certain complex or costly services or drugs (95). Since 2004, G-DRG payment system has been enforced all over the country for most disease fields except for psychiatry (96). Other European countries may use a mix of prospective and retrospective payment models. Some countries may have a more comprehensive payment system that includes DRGs as part of a broader framework. The application of DRGs in Europe may vary by country. Some countries use DRGs mainly in hospitals, while others may extend their use to other healthcare settings. Meanwhile, other countries in the Asia-Pacific region are also formulating and implementing DRG systems to adapt to the ever-changing healthcare environment. This may involve adopting international standard DRG systems or making adjustments and improvements based on the specific healthcare needs of each country. It is important to note that healthcare systems and policies are dynamic, and changes may occur over time. Therefore, the specifics of DRG practices in different countries or regions may evolve based on local needs, policies and healthcare advancements.

In addition, after analyzing the pros and cons of current DRGs practice through DRGKB, we can find useful lessons for countries looking to pilot and further reform DRGs in the future. Medical insurance departments should actively invest manpower and funds to import and modify DRGs, and set up expert groups to make elaborate C&Cs list and grouping method of DRGs, especially in the fields of ICUs, cardiovascular diseases, mental diseases, and trauma. Accordingly, clinical pathways are supposed to establish, including disease process, surgical length of days, etc., so as to improve nursing efficiency and quality.

**Figure 4.**
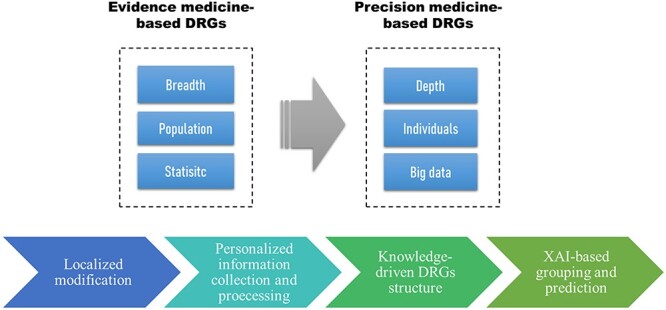
DRGs evolution model and related core concepts.

In the future, different countries need to develop their own local DRG versions with reasonable rate and weight, and each disease group should be refined from the perspective of individual deep data instead of rough population statistics, thereby reducing the outlier rate and financial risk. Hospitals should make good use of big data generated from the hospital systems, so as to implement refined management. Meanwhile, with the improvement of computational power and the completion of large databases, in combination with explainable AI (XAI), data-driven and knowledge-driven-based grouping strategies will become an important trend in the era of big data and precision medicine (97). For example, by enriching grouping nodes through machine learning and deep learning, reasonable DRG grouping and accurate cost prediction can be achieved, reducing upcoding and other behavior motivated by negative incentives. Based on these trends, Figure 4 proposed the precision medicine-based evolution model of DRGs.

## Limitations

At present, we only collected the literature from Pubmed database, while informaton resources from other databases is lacking. For the next update of DRGKB, we will enrich the sources of data. In addition, the literature information is mainly collected and analyzed manually in DRGKB. For the next update, we plan to use natural language processing for literature collection and automate the process to analyze the data more efficiently. In addition, we will also consider establishing a reliability score system for included data (by journal level, research method, etc.).

## Conclusion

In the era of precision medicine and globalization, we need to integrate existing real-world-based data for decision-making. DRGs have been extensively practiced around the world, with mixed results for different reasons. We need to realize that DRGs payment system is not a perfect tool, but are accompanied by various side effects and negative incentives. The knowledgebase established in this study quantifies the performance of DRGs and manually appends textual descriptions and explanations, and categorizes the entries by domain. The knowledgebase establishes a reasonable DRGs practice evaluation structure, which provides an interpretable basis for the follow-up research about decision-making and improvement of DRGs. Complying with the era of value-based medical care, the establishment of this knowledgebase can help DRGs to realize the transition from fee-for-service-based healthcare to an operation pattern based on cost control and quality improvement. At the same time, in the future, researchers should combine DRGs with the latest AI technology and precision medicine paradigm, to maximize the effect of DRGs.

## Supplementary Material

baae046_Supp

## Data Availability

DRGKB is freely available at http://www.sysbio.org.cn/drgkb/.
