# Supplementary material for: DRGKB: a knowledgebase of worldwide diagnosis-related groups’ practices for comparison, evaluation and knowledge-guided application
Source: Database (Oxford). 2024 Jun 6;2024:baae046. doi: 10.1093/database/baae046 (PMC11155695; doi:10.1093/database/baae046)
Supplement: baae046_Supp [file baae046_supp.zip › suppl_data/Appendix 1.docx]

| **Performance** | **Specialties** | **Counts** |
| --- | --- | --- |
| **Low predictability for costs/ALOS** | internal medicine | 1 |
|  | childhood cancer chemotherapy | 1 |
|  | pediatric congenital heart surgery | 1 |
|  | trauma services | 2 |
|  | leukaemia and lymphoma | 1 |
|  | colectomy | 1 |
|  | neonatal intensive care | 2 |
|  | psychiatric illness and substance abuse | 7 |
|  | major reconstructive vascular surgery | 1 |
|  | surgical intensive care | 1 |
|  | acute myocardial infarction | 1 |
|  | cystic fibrosis | 1 |
| **Insufficient consideration about complexity classification** | congenital heart surgery | 1 |
|  | arthroplasty | 3 |
|  | leukaemia and lymphoma | 1 |
|  | diabetes | 1 |
|  | psychiatric illness and substance abuse | 1 |
|  | trauma services | 2 |
|  | pulmonary medicine | 1 |
|  | [neurosurgery](../../../../Software/Youdao/Dict/8.10.3.0/resultui/html/index.html#/javascript:;) | 1 |
| **Low quality of care or insufficient access to medical resources** | hip fracture | 1 |
|  | pelvic organ prolapse | 1 |
|  | endoscopic cardiac surgery | 1 |
|  | service for elderly | 3 |
|  | surgical intensive care | 2 |
|  | gastrointestinal (GI) medicine | 1 |
|  | nephrology | 1 |
|  | major reconstructive vascular surgery | 1 |
| **Inadequate reimbursement** | cardiovascular disease | 5 |
|  | trauma services | 5 |
|  | service for elderly | 2 |
|  | intensive care units | 5 |
|  | nephrology | 2 |

Appendix Table.1 Statistics of specialties distribution of specific indicators
