# Supplementary material for: DRGKB: a knowledgebase of worldwide diagnosis-related groups’ practices for comparison, evaluation and knowledge-guided application
Source: Database (Oxford). 2024 Jun 6;2024:baae046. doi: 10.1093/database/baae046 (PMC11155695; doi:10.1093/database/baae046)
Supplement: baae046_Supp [file baae046_supp.zip › suppl_data/Appendix 2.docx]

| **Specialty** | **Research number** |
| --- | --- |
| cardiovascular disease | 20 |
| intensive care unit | 14 |
| arthroplasty | 10 |
| trauma services | 7 |
| psychiatric illness | 7 |
| neonatal intensive care | 6 |
| acute care | 6 |
| cancer | 6 |
| appendectomy | 4 |
| stroke | 3 |
| substance abuse | 3 |
| pediatrics | 3 |

Appendix Table.2 Statistics of research area
